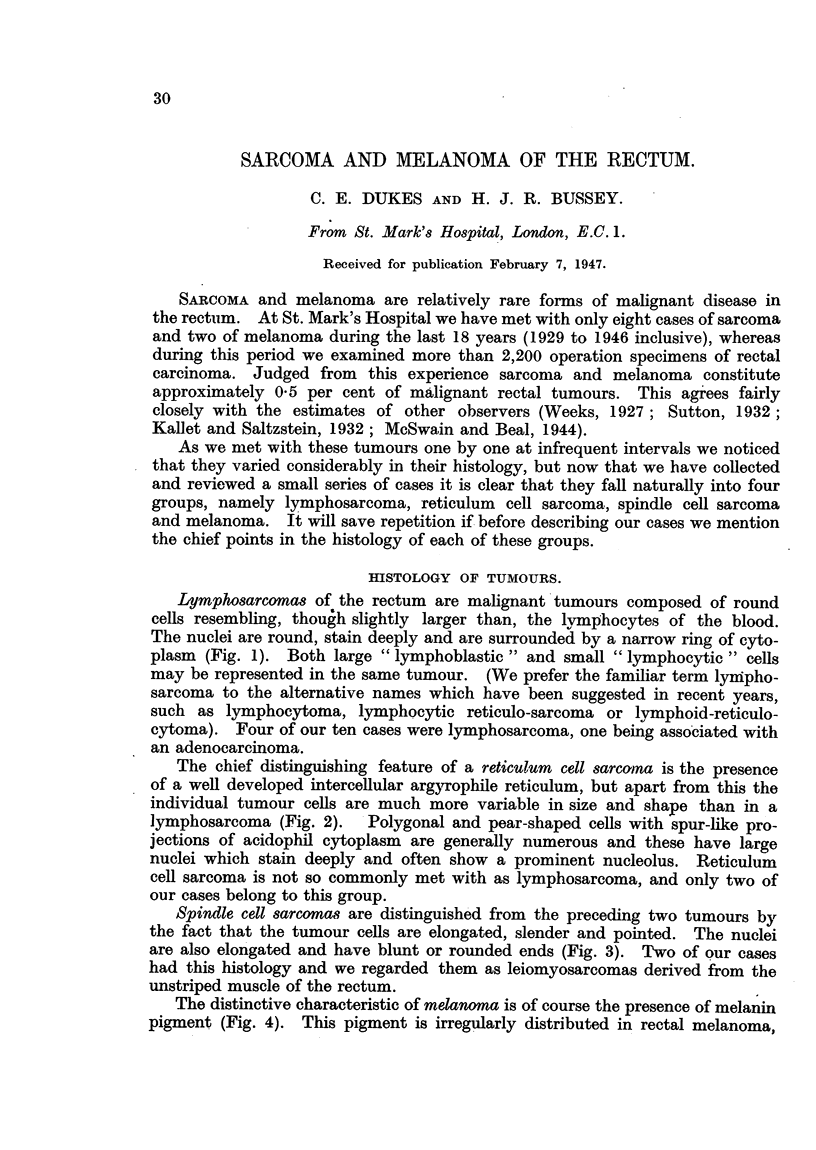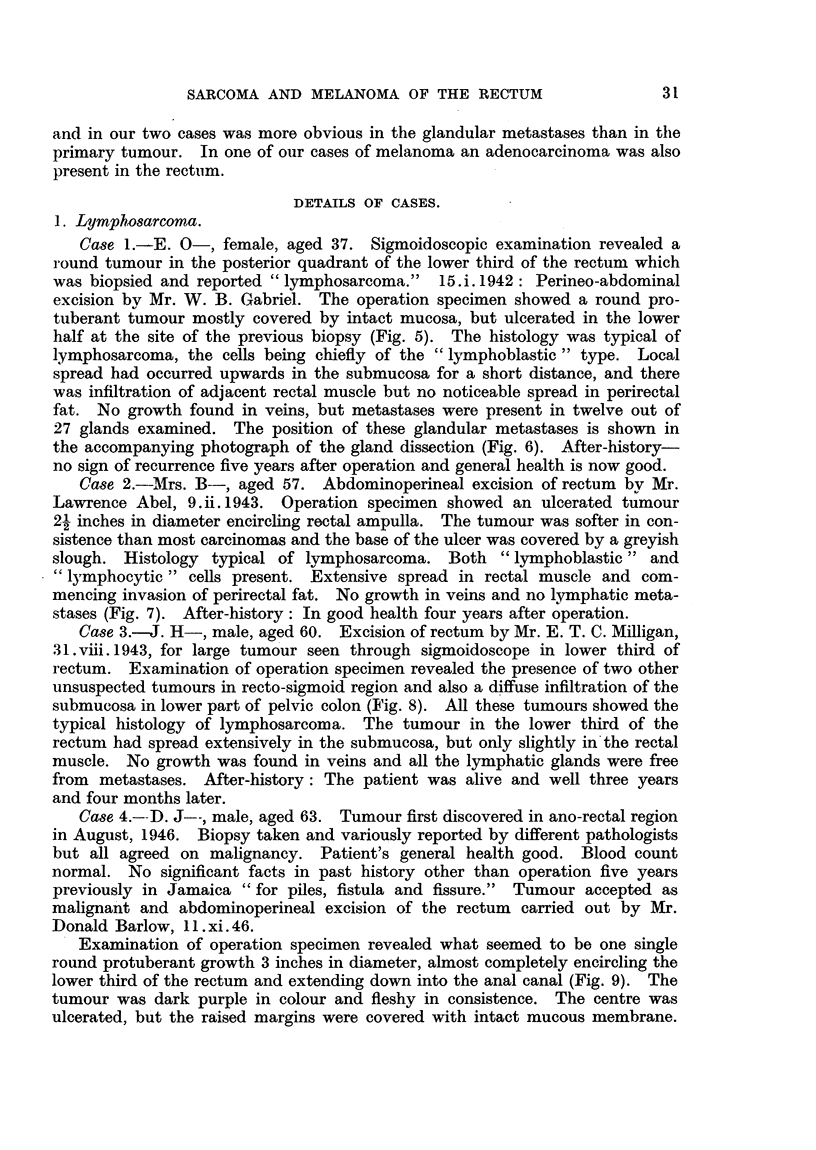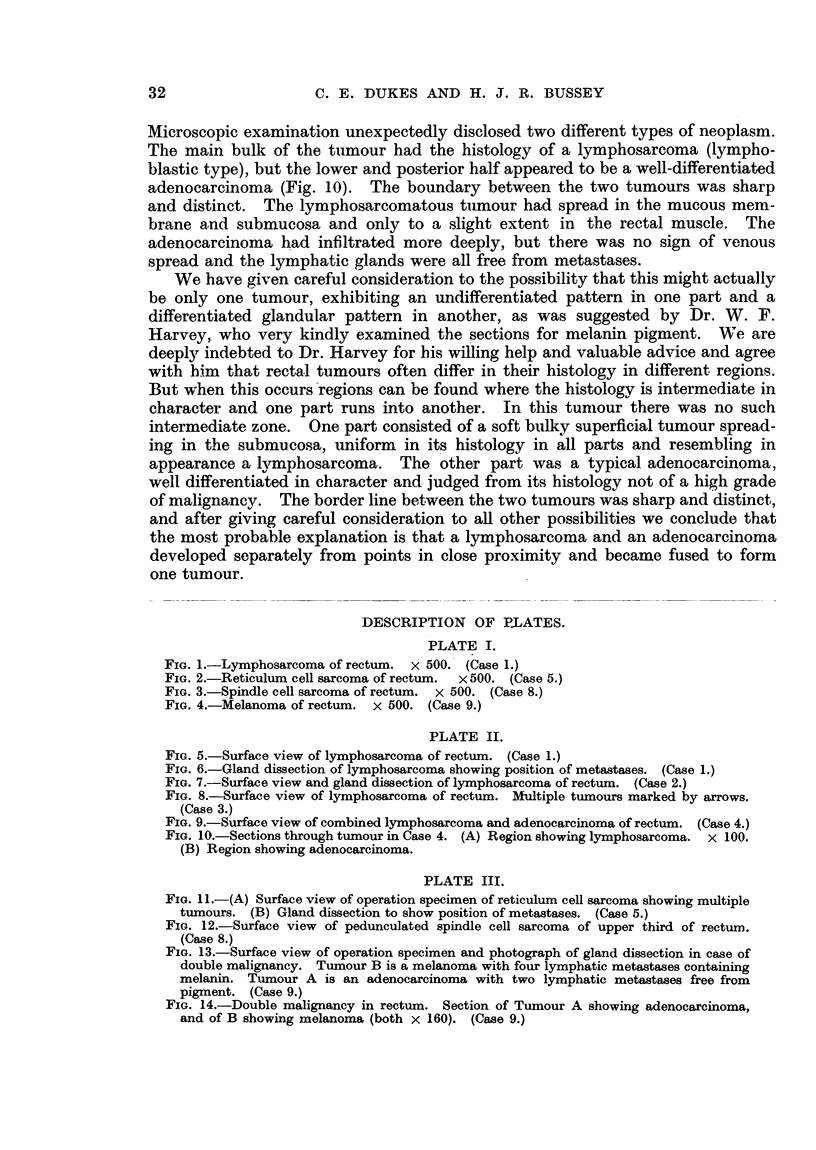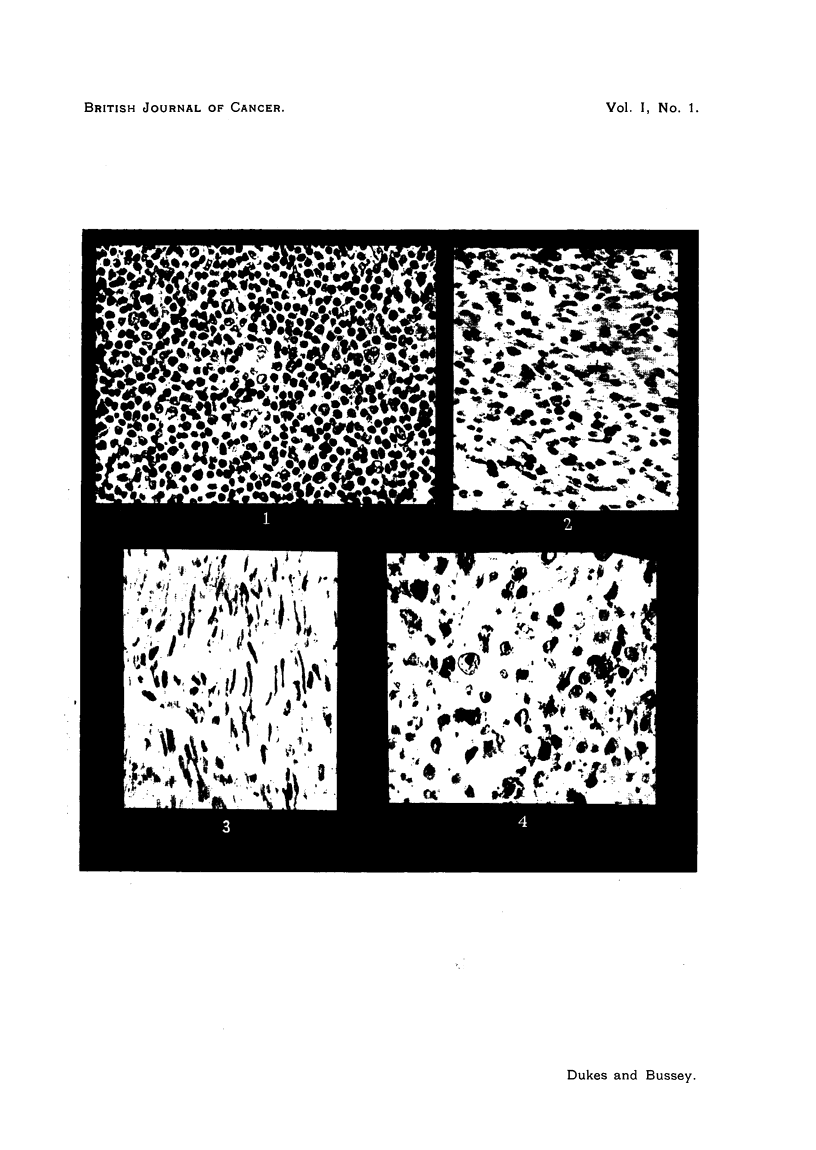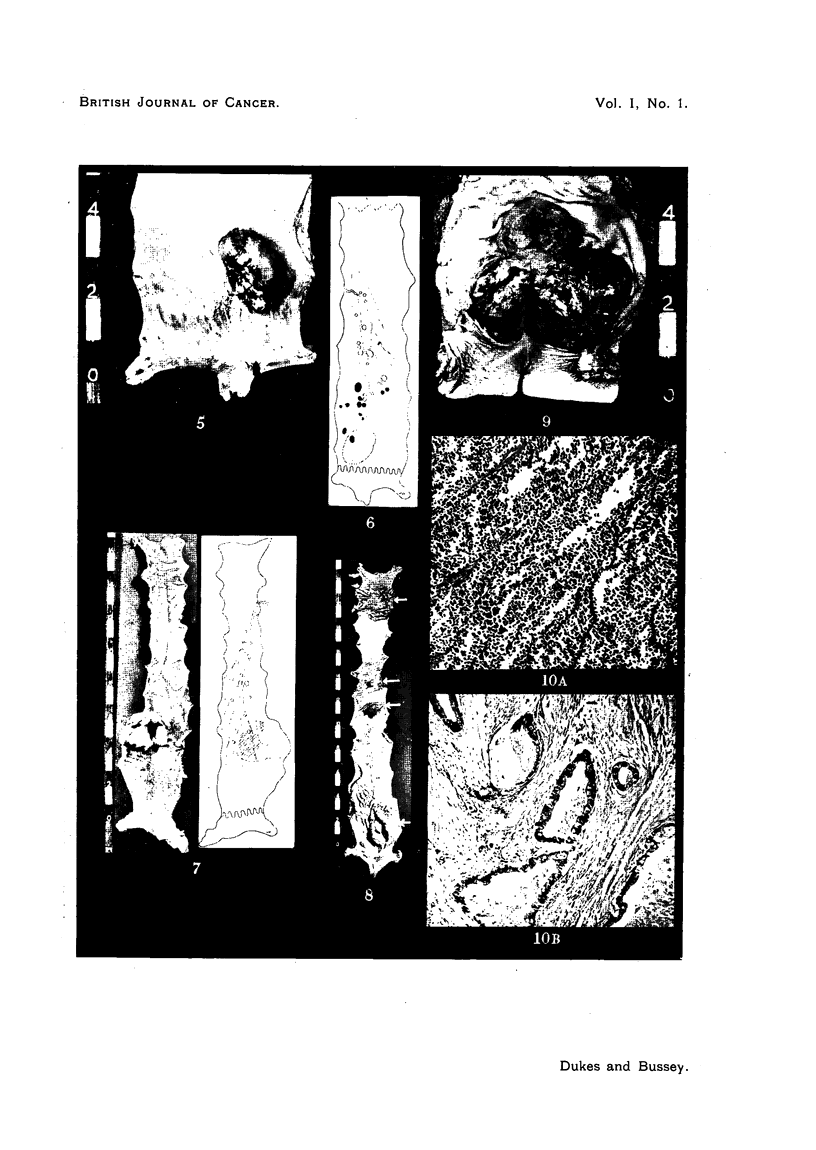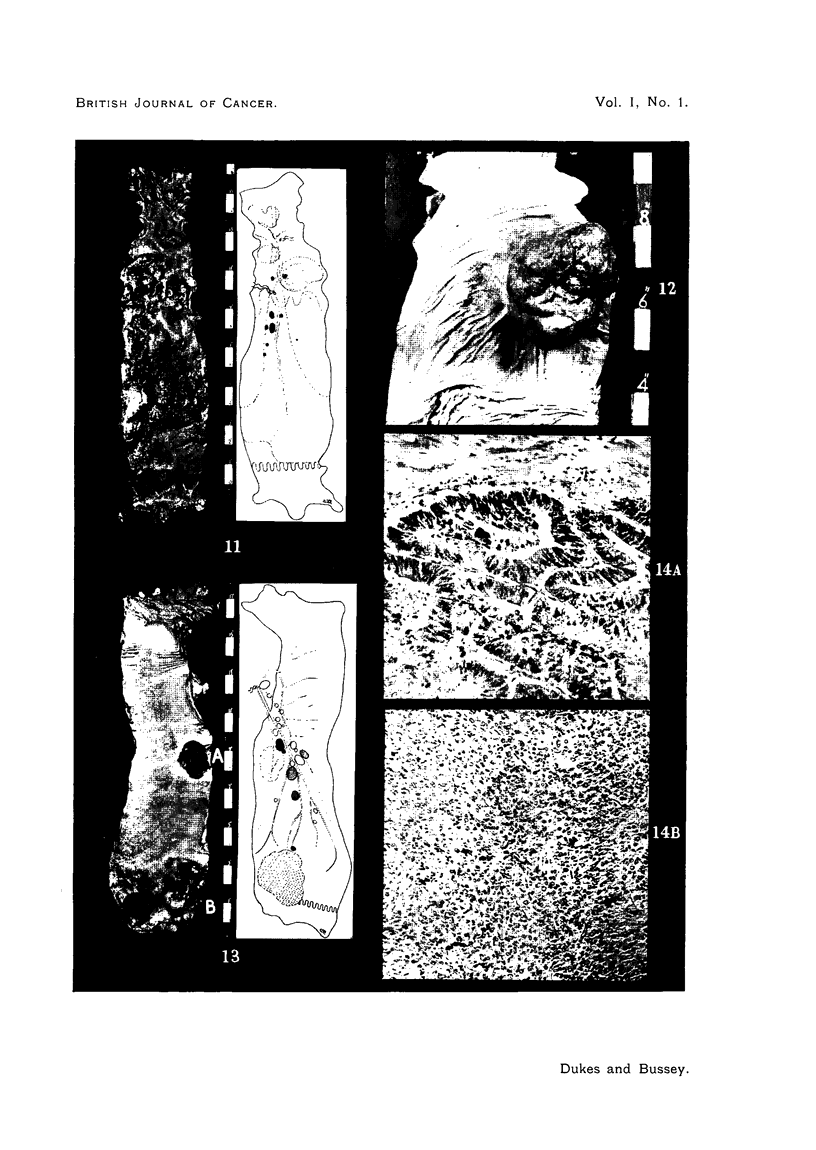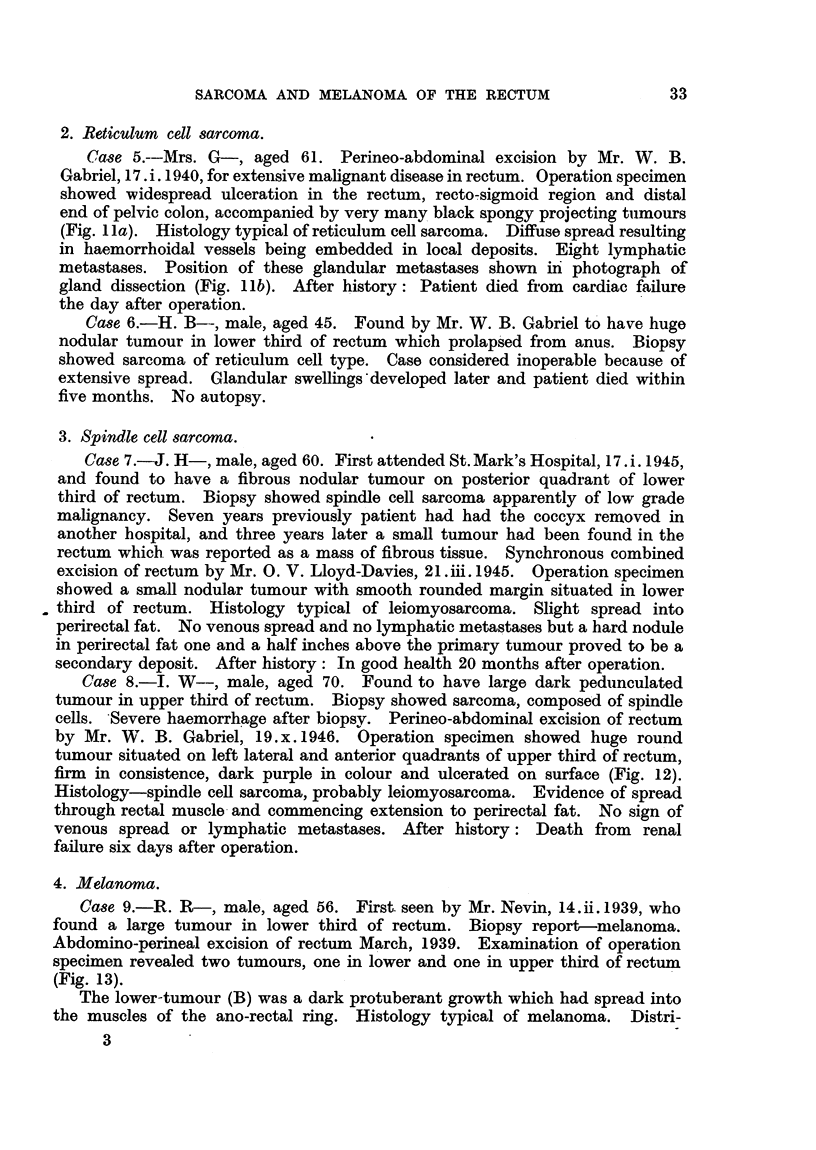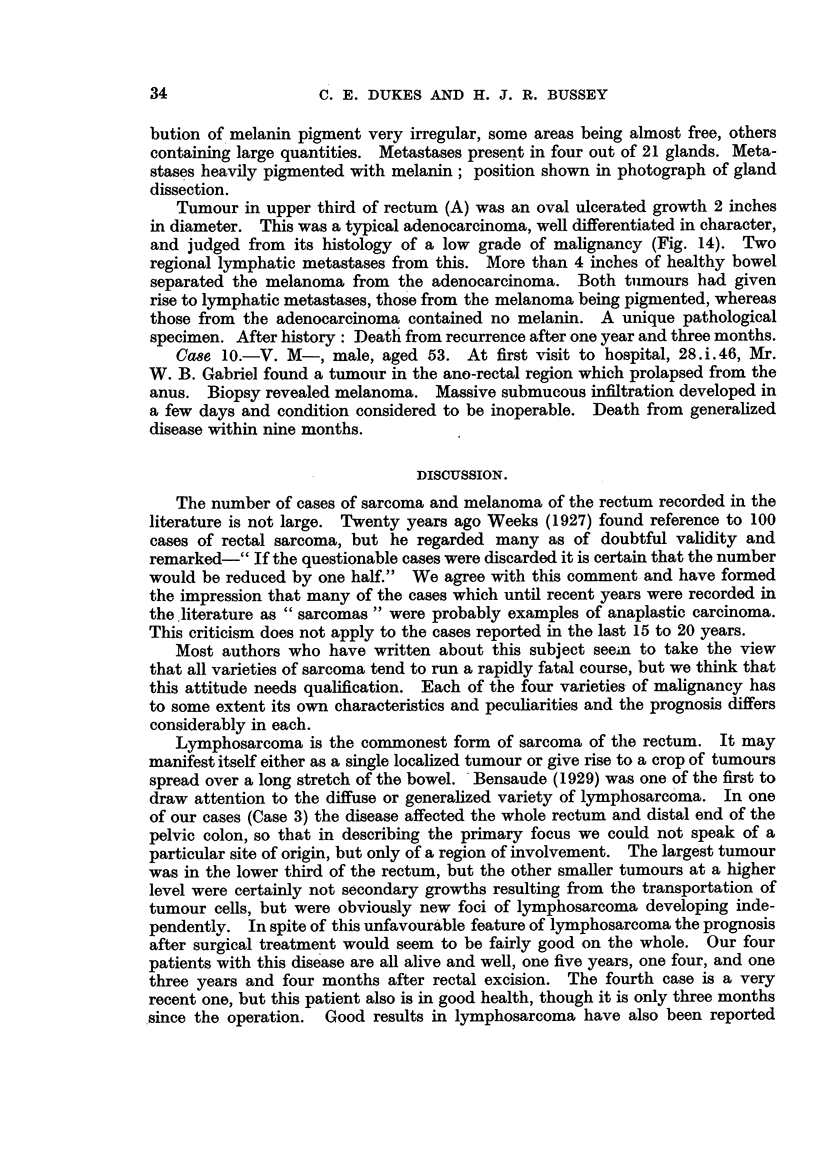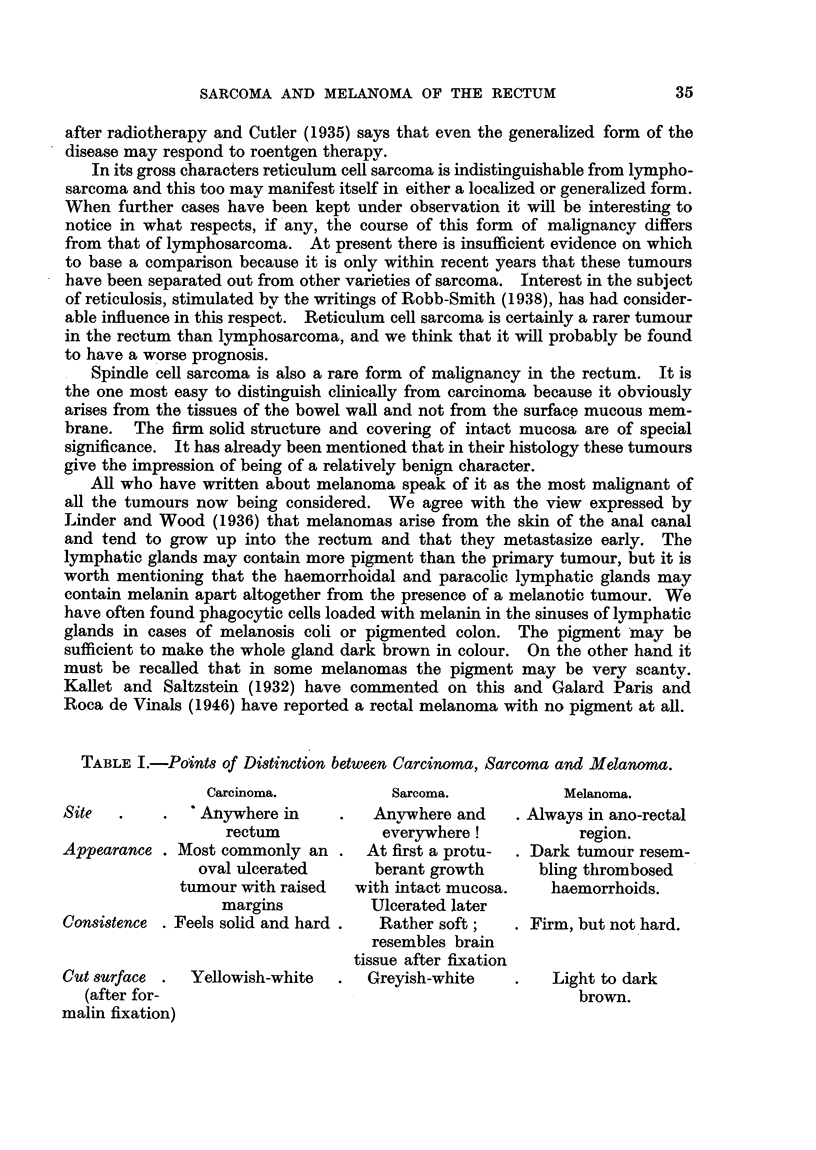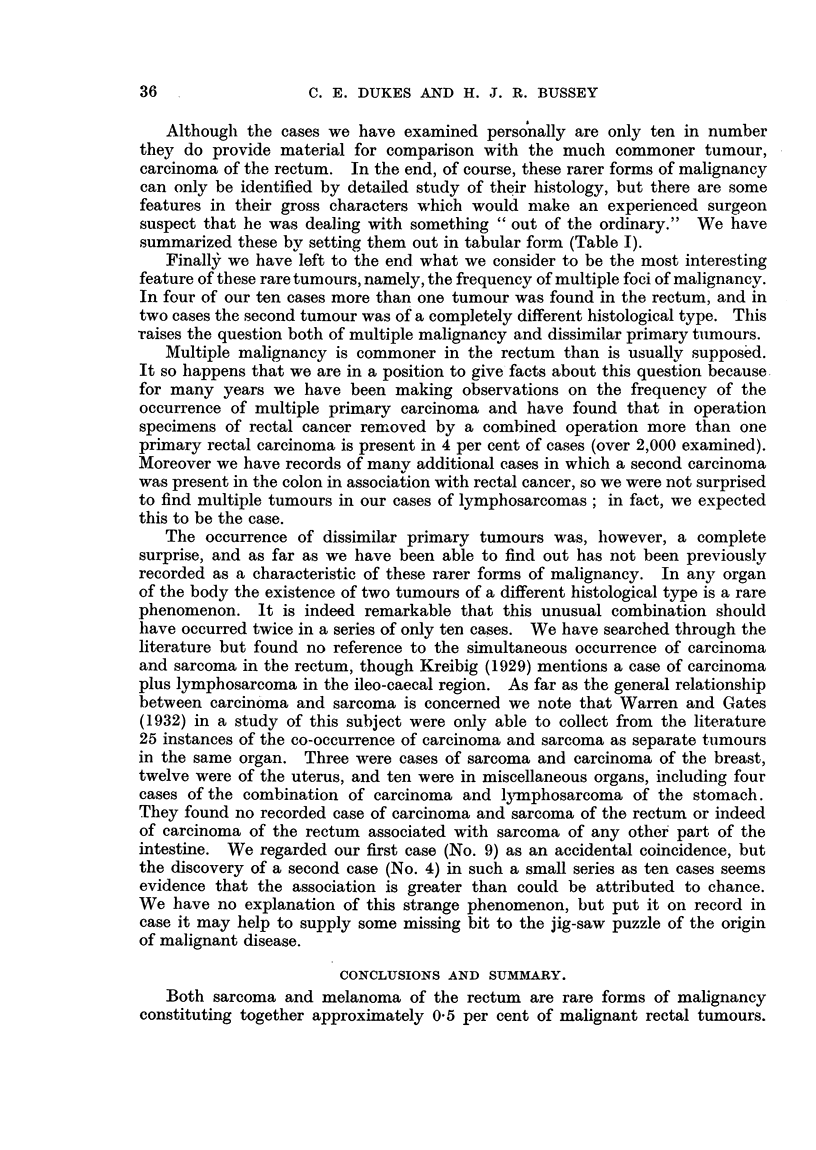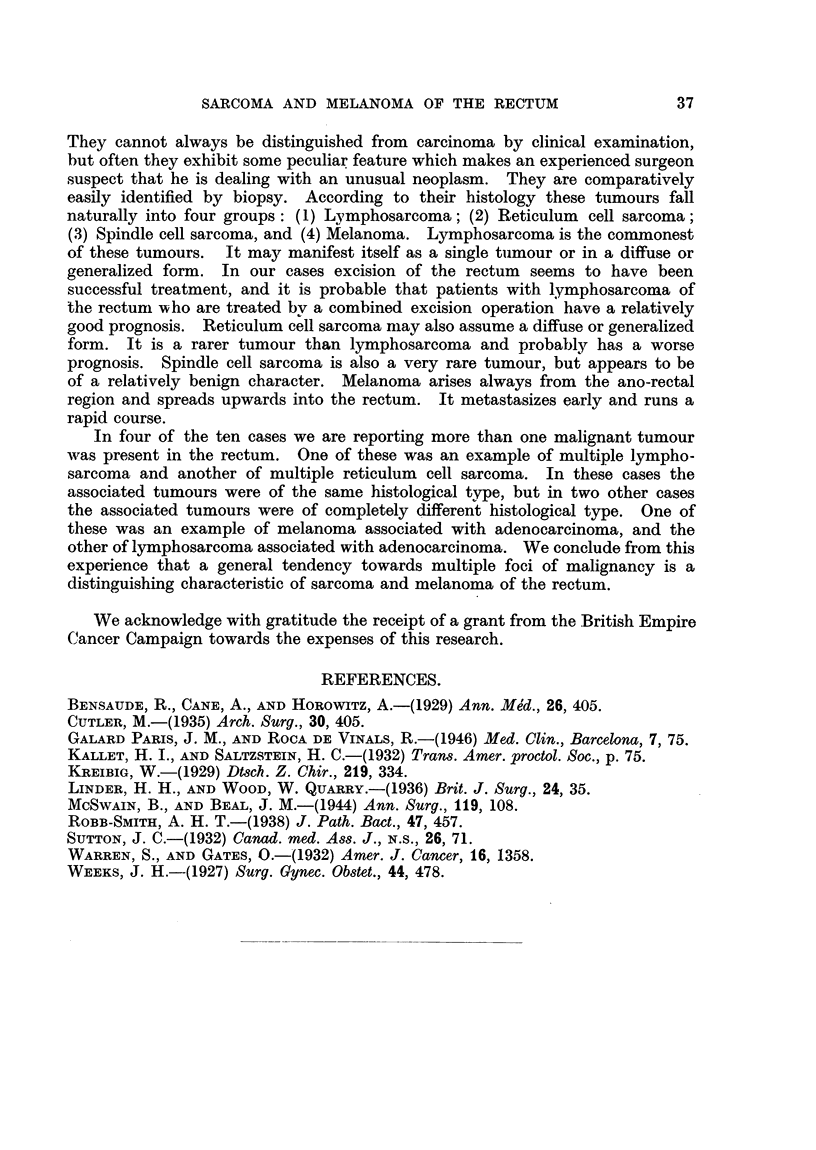# Sarcoma and Melanoma of the Rectum

**DOI:** 10.1038/bjc.1947.4

**Published:** 1947-03

**Authors:** C. E. Dukes, H. J. R. Bussey

## Abstract

**Images:**


### SARCOMA AND MELANOMA OF THE RECTUM.

C. E. DUKES AND H. J. R. BUSSEY.

*From St. Mark's Hospital, London, E.C.* 1.

Received for publication February 7, 1947.

SARCOMA and melanoma are relatively rare forms of malignant disease in the rectum. At St. Mark's Hospital we have met with only eight cases of sarcoma and two of melanoma during the last 18 years (1929 to 1946 inclusive), whereas during this period we examined more than 2,200 operation specimens of rectal carcinoma. Judged from this experience sarcoma and melanoma constitute approximately 0·5 per cent of malignant rectal tumours. This agrees fairly closely with the estimates of other observers (Weeks, 1927 ; Sutton, 1932 ; Kallet and Saltzstein, 1932 ; McSwain and Beal, 1944).

As we met with these tumours one by one at infrequent intervals we noticed that they varied considerably in their histology, but now that we have collected and reviewed a small series of cases it is clear that they fall naturally into four groups, namely lymphosarcoma, reticulum cell sarcoma, spindle cell sarcoma and melanoma. It will save repetition if before describing our cases we mention the chief points in the histology of each of these groups.

### HISTOLOGY OF TUMOURS.

*Lymphosarcomas* of the rectum are malignant tumours composed of round cells resembling, though slightly larger than, the lymphocytes of the blood. The nuclei are round, stain deeply and are surrounded by a narrow ring of cytoplasm (Fig. 1). Both large " lymphoblastic " and small " lymphocytic " cells may be represented in the same tumour. (We prefer the familiar term lymphosarcoma to the alternative names which have been suggested in recent years, such as lymphocytoma, lymphocytic reticulo-sarcoma or lymphoid-reticulocytoma). Four of our ten cases were lymphosarcoma, one being associated with an adenocarcinoma.

The chief distinguishing feature of a *reticulum cell sarcoma* is the presence of a well developed intercellular argyrophile reticulum, but apart from this the individual tumour cells are much more variable in size and shape than in a lymphosarcoma (Fig. 2). Polygonal and pear-shaped cells with spur-like projections of acidophil cytoplasm are generally numerous and these have large nuclei which stain deeply and often show a prominent nucleolus. Reticulum cell sarcoma is not so commonly met with as lymphosarcoma, and only two of our cases belong to this group.

*Spindle cell sarcomas* are distinguished from the preceding two tumours by the fact that the tumour cells are elongated, slender and pointed. The nuclei are also elongated and have blunt or rounded ends (Fig. 3). Two of our cases had this histology and we regarded them as leiomyosarcomas derived from the unstriped muscle of the rectum.

The distinctive characteristic of *melanoma* is of course the presence of melanin pigment (Fig. 4). This pigment is irregularly distributed in rectal melanoma, and in our two cases was more obvious in the glandular metastases than in the primary tumour. In one of our cases of melanoma an adenocarcinoma was also present in the rectum.

### DETAILS OF CASES.

1. *Lymphosarcoma.*

*Case* 1.—E. O—, female, aged 37. Sigmoidoscopic examination revealed a round tumour in the posterior quadrant of the lower third of the rectum which was biopsied and reported "lymphosarcoma." 15.i.1942: Perineo-abdominal excision by Mr. W. B. Gabriel. The operation specimen showed a round protuberant tumour mostly covered by intact mucosa, but ulcerated in the lower half at the site of the previous biopsy (Fig. 5). The histology was typical of lymphosarcoma, the cells being chiefly of the "lymphoblastic" type. Local spread had occurred upwards in the submucosa for a short distance, and there was infiltration of adjacent rectal muscle but no noticeable spread in perirectal fat. No growth found in veins, but metastases were present in twelve out of 27 glands examined. The position of these glandular metastases is shown in the accompanying photograph of the gland dissection (Fig. 6). After-history—no sign of recurrence five years after operation and general health is now good.

*Case* 2.—Mrs. B—, aged 57. Abdominoperineal excision of rectum by Mr. Lawrence Abel, 9.ii.1943. Operation specimen showed an ulcerated tumour 2½ inches in diameter encircling rectal ampulla. The tumour was softer in consistence than most carcinomas and the base of the ulcer was covered by a greyish slough. Histology typical of lymphosarcoma. Both "lymphoblastic" and "lymphocytic" cells present. Extensive spread in rectal muscle and commencing invasion of perirectal fat. No growth in veins and no lymphatic metastases (Fig. 7). After-history: In good health four years after operation.

*Case* 3.—J. H—, male, aged 60. Excision of rectum by Mr. E. T. C. Milligan, 31.viii.1943, for large tumour seen through sigmoidoscope in lower third of rectum. Examination of operation specimen revealed the presence of two other unsuspected tumours in recto-sigmoid region and also a diffuse infiltration of the submucosa in lower part of pelvic colon (Fig. 8). All these tumours showed the typical histology of lymphosarcoma. The tumour in the lower third of the rectum had spread extensively in the submucosa, but only slightly in the rectal muscle. No growth was found in veins and all the lymphatic glands were free from metastases. After-history: The patient was alive and well three years and four months later.

*Case* 4.—D. J—, male, aged 63. Tumour first discovered in ano-rectal region in August, 1946. Biopsy taken and variously reported by different pathologists but all agreed on malignancy. Patient's general health good. Blood count normal. No significant facts in past history other than operation five years previously in Jamaica "for piles, fistula and fissure." Tumour accepted as malignant and abdominoperineal excision of the rectum carried out by Mr. Donald Barlow, 11.xi.46.

Examination of operation specimen revealed what seemed to be one single round protuberant growth 3 inches in diameter, almost completely encircling the lower third of the rectum and extending down into the anal canal (Fig. 9). The tumour was dark purple in colour and fleshy in consistence. The centre was ulcerated, but the raised margins were covered with intact mucous membrane.

Microscopic examination unexpectedly disclosed two different types of neoplasm. The main bulk of the tumour had the histology of a lymphosarcoma (lymphoblastic type), but the lower and posterior half appeared to be a well-differentiated adenocarcinoma (Fig. 10). The boundary between the two tumours was sharp and distinct. The lymphosarcomatous tumour had spread in the mucous membrane and submucosa and only to a slight extent in the rectal muscle. The adenocarcinoma had infiltrated more deeply, but there was no sign of venous spread and the lymphatic glands were all free from metastases.

We have given careful consideration to the possibility that this might actually be only one tumour, exhibiting an undifferentiated pattern in one part and a differentiated glandular pattern in another, as was suggested by Dr. W. F. Harvey, who very kindly examined the sections for melanin pigment. We are deeply indebted to Dr. Harvey for his willing help and valuable advice and agree with him that rectal tumours often differ in their histology in different regions. But when this occurs regions can be found where the histology is intermediate in character and one part runs into another. In this tumour there was no such intermediate zone. One part consisted of a soft bulky superficial tumour spreading in the submucosa, uniform in its histology in all parts and resembling in appearance a lymphosarcoma. The other part was a typical adenocarcinoma, well differentiated in character and judged from its histology not of a high grade of malignancy. The border line between the two tumours was sharp and distinct, and after giving careful consideration to all other possibilities we conclude that the most probable explanation is that a lymphosarcoma and an adenocarcinoma developed separately from points in close proximity and became fused to form one tumour.

---

### DESCRIPTION OF PLATES.

### PLATE I.

FIG. 1.—Lymphosarcoma of rectum. × 500. (Case 1.)

FIG. 2.—Reticulum cell sarcoma of rectum. × 500. (Case 5.)

FIG. 3.—Spindle cell sarcoma of rectum. × 500. (Case 8.)

FIG. 4.—Melanoma of rectum. × 500. (Case 9.)

### PLATE II.

FIG. 5.—Surface view of lymphosarcoma of rectum. (Case 1.)
FIG. 6.—Gland dissection of lymphosarcoma showing position of metastases. (Case 1.)
FIG. 7.—Surface view and gland dissection of lymphosarcoma of rectum. (Case 2.)
FIG. 8.—Surface view of lymphosarcoma of rectum. Multiple tumours marked by arrows. (Case 3.)
FIG. 9.—Surface view of combined lymphosarcoma and adenocarcinoma of rectum. (Case 4.)
FIG. 10.—Sections through tumour in Case 4. (A) Region showing lymphosarcoma. × 100. (B) Region showing adenocarcinoma.

### PLATE III.

FIG. 11.—(A) Surface view of operation specimen of reticulum cell sarcoma showing multiple tumours. (B) Gland dissection to show position of metastases. (Case 5.)
FIG. 12.—Surface view of pedunculated spindle cell sarcoma of upper third of rectum. (Case 8.)
FIG. 13.—Surface view of operation specimen and photograph of gland dissection in case of double malignancy. Tumour B is a melanoma with four lymphatic metastases containing melanin. Tumour A is an adenocarcinoma with two lymphatic metastases free from pigment. (Case 9.)
FIG. 14.—Double malignancy in rectum. Section of Tumour A showing adenocarcinoma, and of B showing melanoma (both × 160). (Case 9.)

1
2
3
4

5

6

9

10A

7

8

10B

8"
6"
12
4"
11
14A
A
B
14B
13

2. *Reticulum cell sarcoma.*

*Case* 5.—Mrs. G—, aged 61. Perineo-abdominal excision by Mr. W. B. Gabriel, 17.i.1940, for extensive malignant disease in rectum. Operation specimen showed widespread ulceration in the rectum, recto-sigmoid region and distal end of pelvic colon, accompanied by very many black spongy projecting tumours (Fig. 11*a*). Histology typical of reticulum cell sarcoma. Diffuse spread resulting in haemorrhoidal vessels being embedded in local deposits. Eight lymphatic metastases. Position of these glandular metastases shown in photograph of gland dissection (Fig. 11*b*). After history: Patient died from cardiac failure the day after operation.

*Case* 6.—H. B—, male, aged 45. Found by Mr. W. B. Gabriel to have huge nodular tumour in lower third of rectum which prolapsed from anus. Biopsy showed sarcoma of reticulum cell type. Case considered inoperable because of extensive spread. Glandular swellings developed later and patient died within five months. No autopsy.

3. *Spindle cell sarcoma.*

*Case* 7.—J. H—, male, aged 60. First attended St. Mark's Hospital, 17.i.1945, and found to have a fibrous nodular tumour on posterior quadrant of lower third of rectum. Biopsy showed spindle cell sarcoma apparently of low grade malignancy. Seven years previously patient had had the coccyx removed in another hospital, and three years later a small tumour had been found in the rectum which was reported as a mass of fibrous tissue. Synchronous combined excision of rectum by Mr. O. V. Lloyd-Davies, 21.iii.1945. Operation specimen showed a small nodular tumour with smooth rounded margin situated in lower third of rectum. Histology typical of leiomyosarcoma. Slight spread into perirectal fat. No venous spread and no lymphatic metastases but a hard nodule in perirectal fat one and a half inches above the primary tumour proved to be a secondary deposit. After history: In good health 20 months after operation.

*Case* 8.—I. W—, male, aged 70. Found to have large dark pedunculated tumour in upper third of rectum. Biopsy showed sarcoma, composed of spindle cells. Severe haemorrhage after biopsy. Perineo-abdominal excision of rectum by Mr. W. B. Gabriel, 19.x.1946. Operation specimen showed huge round tumour situated on left lateral and anterior quadrants of upper third of rectum, firm in consistence, dark purple in colour and ulcerated on surface (Fig. 12). Histology—spindle cell sarcoma, probably leiomyosarcoma. Evidence of spread through rectal muscle and commencing extension to perirectal fat. No sign of venous spread or lymphatic metastases. After history: Death from renal failure six days after operation.

4. *Melanoma.*

*Case* 9.—R. R—, male, aged 56. First seen by Mr. Nevin, 14.ii.1939, who found a large tumour in lower third of rectum. Biopsy report—melanoma. Abdomino-perineal excision of rectum March, 1939. Examination of operation specimen revealed two tumours, one in lower and one in upper third of rectum (Fig. 13).

The lower tumour (B) was a dark protuberant growth which had spread into the muscles of the ano-rectal ring. Histology typical of melanoma. Distribution of melanin pigment very irregular, some areas being almost free, others containing large quantities. Metastases present in four out of 21 glands. Metastases heavily pigmented with melanin ; position shown in photograph of gland dissection.

Tumour in upper third of rectum (A) was an oval ulcerated growth 2 inches in diameter. This was a typical adenocarcinoma, well differentiated in character, and judged from its histology of a low grade of malignancy (Fig. 14). Two regional lymphatic metastases from this. More than 4 inches of healthy bowel separated the melanoma from the adenocarcinoma. Both tumours had given rise to lymphatic metastases, those from the melanoma being pigmented, whereas those from the adenocarcinoma contained no melanin. A unique pathological specimen. After history : Death from recurrence after one year and three months.

*Case* 10.—V. M—, male, aged 53. At first visit to hospital, 28.i.46, Mr. W. B. Gabriel found a tumour in the ano-rectal region which prolapsed from the anus. Biopsy revealed melanoma. Massive submucous infiltration developed in a few days and condition considered to be inoperable. Death from generalized disease within nine months.

### DISCUSSION.

The number of cases of sarcoma and melanoma of the rectum recorded in the literature is not large. Twenty years ago Weeks (1927) found reference to 100 cases of rectal sarcoma, but he regarded many as of doubtful validity and remarked—" If the questionable cases were discarded it is certain that the number would be reduced by one half." We agree with this comment and have formed the impression that many of the cases which until recent years were recorded in the literature as " sarcomas " were probably examples of anaplastic carcinoma. This criticism does not apply to the cases reported in the last 15 to 20 years.

Most authors who have written about this subject seem to take the view that all varieties of sarcoma tend to run a rapidly fatal course, but we think that this attitude needs qualification. Each of the four varieties of malignancy has to some extent its own characteristics and peculiarities and the prognosis differs considerably in each.

Lymphosarcoma is the commonest form of sarcoma of the rectum. It may manifest itself either as a single localized tumour or give rise to a crop of tumours spread over a long stretch of the bowel. Bensaude (1929) was one of the first to draw attention to the diffuse or generalized variety of lymphosarcoma. In one of our cases (Case 3) the disease affected the whole rectum and distal end of the pelvic colon, so that in describing the primary focus we could not speak of a particular site of origin, but only of a region of involvement. The largest tumour was in the lower third of the rectum, but the other smaller tumours at a higher level were certainly not secondary growths resulting from the transportation of tumour cells, but were obviously new foci of lymphosarcoma developing independently. In spite of this unfavourable feature of lymphosarcoma the prognosis after surgical treatment would seem to be fairly good on the whole. Our four patients with this disease are all alive and well, one five years, one four, and one three years and four months after rectal excision. The fourth case is a very recent one, but this patient also is in good health, though it is only three months since the operation. Good results in lymphosarcoma have also been reported after radiotherapy and Cutler (1935) says that even the generalized form of the disease may respond to roentgen therapy.

In its gross characters reticulum cell sarcoma is indistinguishable from lymphosarcoma and this too may manifest itself in either a localized or generalized form. When further cases have been kept under observation it will be interesting to notice in what respects, if any, the course of this form of malignancy differs from that of lymphosarcoma. At present there is insufficient evidence on which to base a comparison because it is only within recent years that these tumours have been separated out from other varieties of sarcoma. Interest in the subject of reticulosis, stimulated by the writings of Robb-Smith (1938), has had considerable influence in this respect. Reticulum cell sarcoma is certainly a rarer tumour in the rectum than lymphosarcoma, and we think that it will probably be found to have a worse prognosis.

Spindle cell sarcoma is also a rare form of malignancy in the rectum. It is the one most easy to distinguish clinically from carcinoma because it obviously arises from the tissues of the bowel wall and not from the surface mucous membrane. The firm solid structure and covering of intact mucosa are of special significance. It has already been mentioned that in their histology these tumours give the impression of being of a relatively benign character.

All who have written about melanoma speak of it as the most malignant of all the tumours now being considered. We agree with the view expressed by Linder and Wood (1936) that melanomas arise from the skin of the anal canal and tend to grow up into the rectum and that they metastasize early. The lymphatic glands may contain more pigment than the primary tumour, but it is worth mentioning that the haemorrhoidal and paracolic lymphatic glands may contain melanin apart altogether from the presence of a melanotic tumour. We have often found phagocytic cells loaded with melanin in the sinuses of lymphatic glands in cases of melanosis coli or pigmented colon. The pigment may be sufficient to make the whole gland dark brown in colour. On the other hand it must be recalled that in some melanomas the pigment may be very scanty. Kallet and Saltzstein (1932) have commented on this and Galard Paris and Roca de Vinals (1946) have reported a rectal melanoma with no pigment at all.

TABLE I.—*Points of Distinction between Carcinoma, Sarcoma and Melanoma.*

| | Carcinoma. | Sarcoma. | Melanoma. |
|---|---|---|---|
| *Site* | Anywhere in rectum | Anywhere and everywhere ! | Always in ano-rectal region. |
| *Appearance* | Most commonly an oval ulcerated tumour with raised margins | At first a protuberant growth with intact mucosa. Ulcerated later | Dark tumour resembling thrombosed haemorrhoids. |
| *Consistence* | Feels solid and hard | Rather soft ; resembles brain tissue after fixation | Firm, but not hard. |
| *Cut surface* (after formalin fixation) | Yellowish-white | Greyish-white | Light to dark brown. |

Although the cases we have examined personally are only ten in number they do provide material for comparison with the much commoner tumour, carcinoma of the rectum. In the end, of course, these rarer forms of malignancy can only be identified by detailed study of their histology, but there are some features in their gross characters which would make an experienced surgeon suspect that he was dealing with something " out of the ordinary." We have summarized these by setting them out in tabular form (Table I).

Finally we have left to the end what we consider to be the most interesting feature of these rare tumours, namely, the frequency of multiple foci of malignancy. In four of our ten cases more than one tumour was found in the rectum, and in two cases the second tumour was of a completely different histological type. This raises the question both of multiple malignancy and dissimilar primary tumours.

Multiple malignancy is commoner in the rectum than is usually supposed. It so happens that we are in a position to give facts about this question because for many years we have been making observations on the frequency of the occurrence of multiple primary carcinoma and have found that in operation specimens of rectal cancer removed by a combined operation more than one primary rectal carcinoma is present in 4 per cent of cases (over 2,000 examined). Moreover we have records of many additional cases in which a second carcinoma was present in the colon in association with rectal cancer, so we were not surprised to find multiple tumours in our cases of lymphosarcomas ; in fact, we expected this to be the case.

The occurrence of dissimilar primary tumours was, however, a complete surprise, and as far as we have been able to find out has not been previously recorded as a characteristic of these rarer forms of malignancy. In any organ of the body the existence of two tumours of a different histological type is a rare phenomenon. It is indeed remarkable that this unusual combination should have occurred twice in a series of only ten cases. We have searched through the literature but found no reference to the simultaneous occurrence of carcinoma and sarcoma in the rectum, though Kreibig (1929) mentions a case of carcinoma plus lymphosarcoma in the ileo-caecal region. As far as the general relationship between carcinoma and sarcoma is concerned we note that Warren and Gates (1932) in a study of this subject were only able to collect from the literature 25 instances of the co-occurrence of carcinoma and sarcoma as separate tumours in the same organ. Three were cases of sarcoma and carcinoma of the breast, twelve were of the uterus, and ten were in miscellaneous organs, including four cases of the combination of carcinoma and lymphosarcoma of the stomach. They found no recorded case of carcinoma and sarcoma of the rectum or indeed of carcinoma of the rectum associated with sarcoma of any other part of the intestine. We regarded our first case (No. 9) as an accidental coincidence, but the discovery of a second case (No. 4) in such a small series as ten cases seems evidence that the association is greater than could be attributed to chance. We have no explanation of this strange phenomenon, but put it on record in case it may help to supply some missing bit to the jig-saw puzzle of the origin of malignant disease.

### CONCLUSIONS AND SUMMARY.

Both sarcoma and melanoma of the rectum are rare forms of malignancy constituting together approximately 0·5 per cent of malignant rectal tumours.

They cannot always be distinguished from carcinoma by clinical examination, but often they exhibit some peculiar feature which makes an experienced surgeon suspect that he is dealing with an unusual neoplasm. They are comparatively easily identified by biopsy. According to their histology these tumours fall naturally into four groups: (1) Lymphosarcoma; (2) Reticulum cell sarcoma; (3) Spindle cell sarcoma, and (4) Melanoma. Lymphosarcoma is the commonest of these tumours. It may manifest itself as a single tumour or in a diffuse or generalized form. In our cases excision of the rectum seems to have been successful treatment, and it is probable that patients with lymphosarcoma of the rectum who are treated by a combined excision operation have a relatively good prognosis. Reticulum cell sarcoma may also assume a diffuse or generalized form. It is a rarer tumour than lymphosarcoma and probably has a worse prognosis. Spindle cell sarcoma is also a very rare tumour, but appears to be of a relatively benign character. Melanoma arises always from the ano-rectal region and spreads upwards into the rectum. It metastasizes early and runs a rapid course.

In four of the ten cases we are reporting more than one malignant tumour was present in the rectum. One of these was an example of multiple lymphosarcoma and another of multiple reticulum cell sarcoma. In these cases the associated tumours were of the same histological type, but in two other cases the associated tumours were of completely different histological type. One of these was an example of melanoma associated with adenocarcinoma, and the other of lymphosarcoma associated with adenocarcinoma. We conclude from this experience that a general tendency towards multiple foci of malignancy is a distinguishing characteristic of sarcoma and melanoma of the rectum.

We acknowledge with gratitude the receipt of a grant from the British Empire Cancer Campaign towards the expenses of this research.

### REFERENCES.

Bensaude, R., Cane, A., and Horowitz, A.—(1929) *Ann. Méd.*, **26**, 405.
Cutler, M.—(1935) *Arch. Surg.*, **30**, 405.
Galard Paris, J. M., and Roca de Vinals, R.—(1946) *Med. Clin., Barcelona*, **7**, 75.
Kallet, H. I., and Saltzstein, H. C.—(1932) *Trans. Amer. proctol. Soc.*, p. 75.
Kreibig, W.—(1929) *Dtsch. Z. Chir.*, **219**, 334.
Linder, H. H., and Wood, W. Quarry.—(1936) *Brit. J. Surg.*, **24**, 35.
McSwain, B., and Beal, J. M.—(1944) *Ann. Surg.*, **119**, 108.
Robb-Smith, A. H. T.—(1938) *J. Path. Bact.*, **47**, 457.
Sutton, J. C.—(1932) *Canad. med. Ass. J.*, n.s., **26**, 71.
Warren, S., and Gates, O.—(1932) *Amer. J. Cancer*, **16**, 1358.
Weeks, J. H.—(1927) *Surg. Gynec. Obstet.*, **44**, 478.